# The role of carbon nanotubes in enhanced charge storage performance of VSe_2_: experimental and theoretical insight from DFT simulations†

**DOI:** 10.1039/d0ra06773c

**Published:** 2020-08-27

**Authors:** Sree Raj K. A., Afsal S. Shajahan, Brahmananda Chakraborty, Chandra Sekhar Rout

**Affiliations:** Centre for Nano and Material Sciences Jain Global Campus, Jakkasandra, Ramanagaram Bangalore-562112 India r.chandrasekhar@jainuniversity.ac.in csrout@gmail.com; High Pressure and Synchrotron Radiation Physics Division, Bhabha Atomic Research Centre Trombay Mumbai 400085 India; Homi Bhabha National Institute Mumbai 400094 India brahma@barc.gov.in

## Abstract

Herein, we report the hybrid structure of metallic VSe_2_ and multi-walled carbon nanotube (MWCNT) based hybrid materials for high performance energy storage and high power operation applications. The dominance of capacitive energy storage performance behaviour of VSe_2_/MWCNT hybrids is observed. A symmetric supercapacitor cell device fabricated using VSe_2_/80 mg MWCNT delivered a high energy density of 46.66 W h kg^−1^ and a maximum power density of 14.4 kW kg^−1^ with a stable cyclic operation of 87% after 5000 cycles in an aqueous electrolyte. Using density functional theory calculations we have presented structural and electronic properties of the hybrid VSe_2_/MWCNT structure. Enhanced states near the Fermi level and higher quantum capacitance for the hybrid structure contribute towards higher energy and power density for the nanotube/VSe_2_.

## Abstract 2

Hybrid structure of VSe_2_ and multiwalled carbon nanotube for high performance supercapacitor applications.

## Graphical abstract

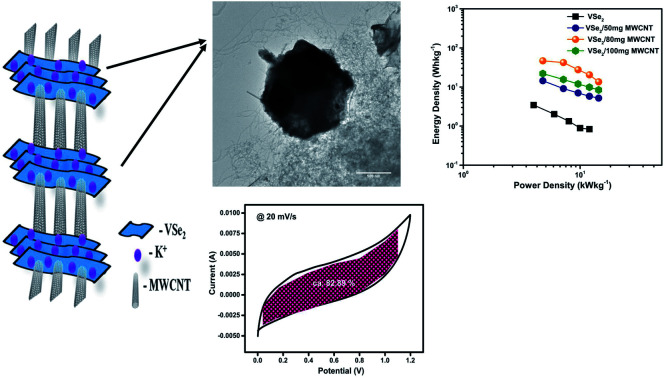

## 1 Introduction

The growing demands for energy resources and the limitations of fossil fuels have stimulated intense research on alternative high-performance energy storage devices. Batteries and supercapacitors are the most favourable devices for these purposes. Batteries can store more energy than supercapacitors but the faradaic reaction to store energy in batteries hinders their use in high power operations.^1^ Unlike batteries, supercapacitors have higher power density which helps them to accumulate and provide more energy for a shorter period compared to batteries.^2^ Their high cycling life and reversibility also give them an edge over other energy storage devices.^3^ According to the energy storage mechanism, supercapacitors can be categorized into three types *i.e.* (1) electric double-layer capacitors (EDLCs), (2) pseudocapacitors and (3) battery like capacitors.^4,5^ In a conventional EDLC, charge storage occurs due to the formation of an electric double layer at the electrode–electrolyte interfaces.^6^ The occurrence of a faradaic electron transfer in the energy storage makes the device a battery-like supercapacitor.^7^ In the case of pseudocapacitors, rapid faradaic reactions occur at the surface or near-surface of the electrode and there is no solid-state diffusion like batteries.^8,9^ Transition metal dichalcogenides (TMDs) are rigorously expedited 2D materials for electrochemical energy storage applications.^10,11^ Properties like large surface area, polytypic structure (2H and 1T), easiness to functionalize, flexibility toward the controlled strain and variable oxidation state make them the most desirable candidates for both EDLC and pseudocapacitors based energy storage devices.^12–14^ Among TMDs, 1T-VSe_2_ is one of the attractive electrode material having high electrical conductivity and surface area.^15,16^

The basic structure of 1T-VSe_2_ (ref. 17) is the octahedral vanadium atom coordination in a tetragonal symmetry with an offset arrangement of layers held together by weak van der Waal force. This layered structure, highly active edges and basal sites bestowed prepossessing energy storage properties to VSe_2_.^18^ 1T structure of VSe_2_ evinced metallic behaviour in nature.^19^ The unpaired d electron of vanadium is responsible for the high electronic conductivity.^20^ The van der Waal gaps in metallic TMDs can electrochemically intercalate cations with higher efficiency and plays important role in exhibiting superior charge storage properties.^21^ However, the high Gibbs's free energy makes 1T-VSe_2_ less stable.^21^ Interestingly, with the help of highly porous carbonaceous materials such as graphene and CNTs, it is possible to obtain high stability in long cycle operations along with enhanced electrochemical properties due soared active sites and enhanced electronic conductivity.^22–24^ Behera and co-workers successfully synthesized VSe_2_/RGO composites for supercapacitor applications with an energy density of 212 W h kg^−1^ at a power density of 0.9 kW kg^−1^.^25^ The multi-walled carbon nanotube (MWCNT) can improve the electrochemical activity of 1T-VSe_2_ due to its large surface area, high electronic conductivity, better chemical stability and excellent flexibility.^26,27^ Wu *et al.* fabricated VSe_2_/CNT based in-plane supercapacitor which although improves the stability of the material but there is no noticeable increase in capacitance.^28^ Coexistence of improved energy density, power density and cyclic stability of 1T-VSe_2_ based electrodes is very much required for the commercial viability.

Here, we propose a one-step hydrothermal technique to synthesize VSe_2_/MWNTs hybrid electrodes with enhanced ionic conductivity, long cycle life and very high energy and power density. We have provided a comprehensive understanding of the energy storage mechanism involved in VSe_2_/MWCNTs with different concentration of CNTs suitable for commercial application. Theoretical simulations have been carried out to support experimental findings from electronic properties, bonding mechanism and quantum capacitance of the hybrid structure as well as diffusion and charge transfer performance of the electrolyte ions.

## 2 Experimental section

### 2.1 Synthesis of VSe_2_/MWCNT composites

High purity MWCNTs (<5% impurities, 1–10 μm length and 3–15 number of walls) was purchased from PlasmaChem GmbH, Berlin. The nanotubes were functionalized using an acidic solution of H_2_SO_4_ and HNO_3_ mixed together in a 3 : 1 ratio. The VSe_2_/MWCNT hybrids are synthesised by following the one-step hydrothermal method. 1 mM of ammonium metavanadate (NH_4_VO_3_), 2 mM of selenium dioxide (SeO_2_), 5 ml formic acid and different concentrations of functionalized multi-walled carbon nanotube (MWCNT) were added into 35 ml distilled water and stirred until a uniform solution was obtained. The above solution then transferred into a Teflon lined autoclave and heated up to 200 °C for 24 h. After the autoclave reaches room temperature, the precipitate is washed in distilled water and ethanol, followed by drying in ambient conditions. Pristine VSe_2_ also synthesised in the same method without the addition of nanotubes.

### 2.2 Material characterization

The morphological and structural characterisations of VSe_2_/MWCNT composites were carried out using Field Emission Scanning Electron Microscope (FESEM, JEOL JSM-7100F, JEOL Ltd., Singapore, with maximum operating accelerating voltage as 30 kV), Transmission Electron Microscope (TEM, TALOS F200S G2 with 200 kV, FEG, CMOS Camera 4k × 4k) and X-ray Diffraction (XRD) (Rigaku Ultima IV X-ray diffractometer having Ni-filter for Cu Kα radiation (wavelength, *λ* = 0.1541 nm)). FESEM has also been used to colour map the elements present in the material. The surface area measurements were done by BET surface area analyzer (Belsorp max, Japan).

### 2.3 Electrochemical measurements

The electrochemical measurements were studied using Wuhan Corrtest electrochemical workstation version 5.3 using a Swagelok cell in two electrode configuration with 0.5 M K_2_SO_4_ as an aqueous electrolyte. Electrochemical impedance spectroscopy (EIS) for the symmetric device was performed within the frequency range between 0.05 Hz and 100 kHz with operating ac field amplitude of 5 mV.

### 2.4 Computational details

We have used plane wave based Density Functional Theory (DFT) code VASP^29–32^ with PAW-GGA as exchange–correlation functional^49^ for the simulations. The cutoff energy is considered as 500 eV and the Brillouin zone is integrated employing a Monkhorst–Pack mesh of 7 × 7 × 1 *k*-points for (001) plane of VSe_2_ and hybrid VSe_2_/CNT (carbon nanotube). To describe the weak van der Waals forces between VSe_2_ and CNT, we have considered Grimme DFT-D2 (ref. 33) dispersion scheme.

## 3 Results and discussions

### 3.1 Morphological and structural characterization of VSe_2_/MWCNT hybrid sheets

**Fig. 1:**
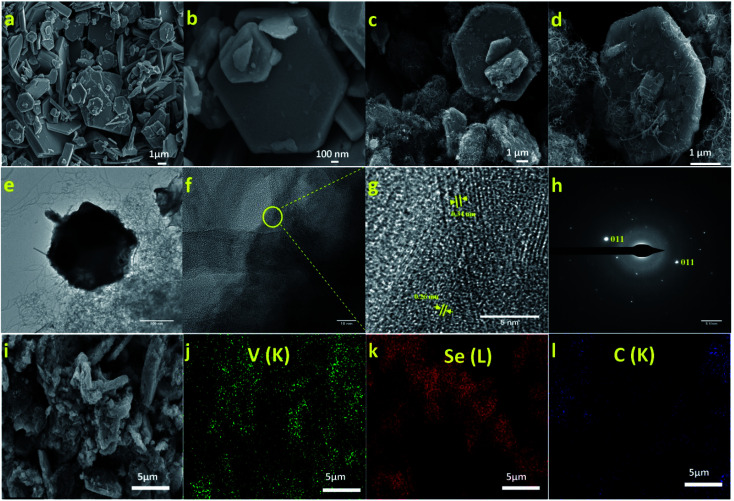
(a) Low and (b) high magnification FESEM images for of pristine VSe_2_. (c) Low and (d) high magnification FESEM images VSe_2_/80 mg MWCNT composite, (e–g) low and high resolution TEM image of VSe_2_/80 mg MWCNT composite, (h) SAED pattern of VSe_2_/80 mg MWCNT composite and (i–l) elemental mapping of VSe_2_/80 mg MWCNT.

The morphology of VSe_2_/MWCNT and pristine VSe_2_ were studied through field emission scanning electron microscopy (FESEM) which is shown in Fig. 1a–d. FESEM images revealed that the pristine VSe_2_ forms as hexagonal nanosheets. In VSe_2_/MWCNT composite, VSe_2_ exhibits a similar hexagonal morphology with interconnected MWCNTs uniformly. From Fig. 1c and d it is observed that carbon nanotubes extend from one to other hexagonal VSe_2_ flake throughout the material. Also, similar structure formation is observed in the other composites with different concentration of MWCNTs (Fig. S1a–f of ESI†). In the case of VSe_2_/100 mg MWCNT hybrid, agglomeration of CNTs was observed (Fig. S1e and f of ESI†). The energy-dispersive X-ray spectroscopy (EDS) suggests the presence of V and Se in the 1T-VSe_2_, while VSe_2_/80 mg MWCNT shows a C peak which belongs to MWCNT (Fig. S2 ESI†). Further, elementary mapping of the VSe_2_/MWCNT composite is shown in Fig. 1i–l, which demonstrates the uniform distribution of the V, Se and C elements. The High-Resolution Transmission Microscope (HRTEM) images of VSe_2_/80 mg MWCNT hybrid is given in Fig. 1e. The HRTEM analysis showcases the concatenated structure of VSe_2_ and MWCNT (Fig. 1f). The lattice fringes measured in the intersection of VSe_2_ and MWCNT are having a *d* spacing of 0.26 nm which belongs to the (011) plane of VSe_2_ and the other fringe with *d* spacing of 0.34 nm belongs to the (002) plane of MWCNT (Fig. 1g). The SAED pattern of VSe_2_/80 mg MWCNT is given in Fig. 1h.

The XRD patterns of VSe_2_ and VSe_2_/MWCNT composites are shown in Fig. S3 ESI.† All the peaks of VSe_2_ and VSe_2_/MWCNT composites are facsimiled with the JCPDS card number 89-1641. The XRD patterns of VSe_2_ and VSe_2_/MWCNT revealed the formation of the metallic VSe_2_, but in the case of hybrids with a high concentration of nanotubes, low content of vanadium oxide coexists. During the synthesis process, the functional groups attached to the CNT surface facilitate the grafting of this low content of vanadium oxide layer on it.^34^ The broadening of (002) plane in VSe_2_/100 mg MWCNT may be due to the increased strain in VSe_2_ sheets. The interconnection of VSe_2_ sheets by MWCNT causes a strain which leads to lattice distortion and peak broadening.^35^ The growth and slight shift of (002) peak and diminution of highly intense (011) peak can be explained by the possible increase of interlayer spacing between the VSe_2_ layers.^25,36^ In the case of the hybrids VSe_2_/50 mg MWCNT and VSe_2_/80 mg MWCNT, prominent peaks of MWCNT are not observed due to formation of high crystalline metallic VSe_2_ and its dominance over CNTs. In the case of the VSe_2_/100 mg MWCNT, a less intensified broad (002) peak of MWCNT is observed around 26°. The specific surface area of metallic VSe_2_ and VSe_2_/80 mg MWCNT was calculated using Brunauer–Emmett–Teller (BET) analysis. The 1T-VSe_2_ shows a specific surface area of 3.5478 m^2^ g^−1^ and the VSe_2_/80 mg MWCNT composite exhibits an enhanced specific surface area of 93.047 m^2^ g^−1^ (Fig. S4 of ESI†).

The charge storage mechanism in a supercapacitor electrode involves three major contributors: (1) the formation of an electric double layer in the electrode–electrolyte interface of the cell, (2) pseudocapacitive process arising from a non-faradaic surface redox reaction or diffusion free intercalation of ions into layers of the electrode material and (3) faradaic reaction arising from the intercalation mechanism. The first two mechanisms convoluted to the capacitive type storage and the third mechanism contributes to diffusive type charge storage. The faradaic and non-faradaic components are given in eqn (1) and (2) respectively.^37^

1 VSe_2_ + K^+^ + e^−^ ⇌ VSe − SeK

2 (VSe_2_)_surface_ + K^+^ + e^−^ ⇌ (VSe_2_^−^ − K^+^)_surfcae_

The charge storage contributions from both faradaic and capacitive components can be calculated from the CV curves by using the Power's law (eqn (3)):^38^

3 *i* = *aυ*^*b*^

According to the Power's law, *i* (A) is the current at a particular voltage, *υ* (V s^−1^) is the scan rate *a* and *b* are the two adjustable parameters.^38^ The *b* parameter can be obtained from the slop after plotting log *i* against log *υ* at a particular potential (*V*).^8^ Generally, if the *b* value is equal to 1 then its shows a diffusion free capacitive behaviour.^39^ If the *b* value equal to 1/2 impute an ideal diffusion dependant faradaic contribution which satisfies Cortell's equation *i* = *aυ*^1/2^.^38^ Therefore, current value *i* (A) at a fixed potential *V* from CV can be written as the sum of surface redox reactions and diffusion dependent faradaic reactions (eqn (4a) and (4b))

4a *i*(*V*) = *a*_1_*υ* + *a*_2_*υ*^1/2^

4b
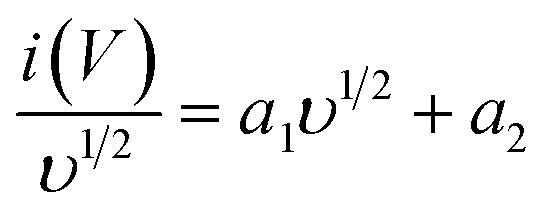


The values of *a*_1_ and *a*_2_ can be determined from the slope and intercept of the *i*(*V*)/*υ*^1/2^*vs. υ*^1/2^ plot at specific potential values.^40^

**Fig. 2:**
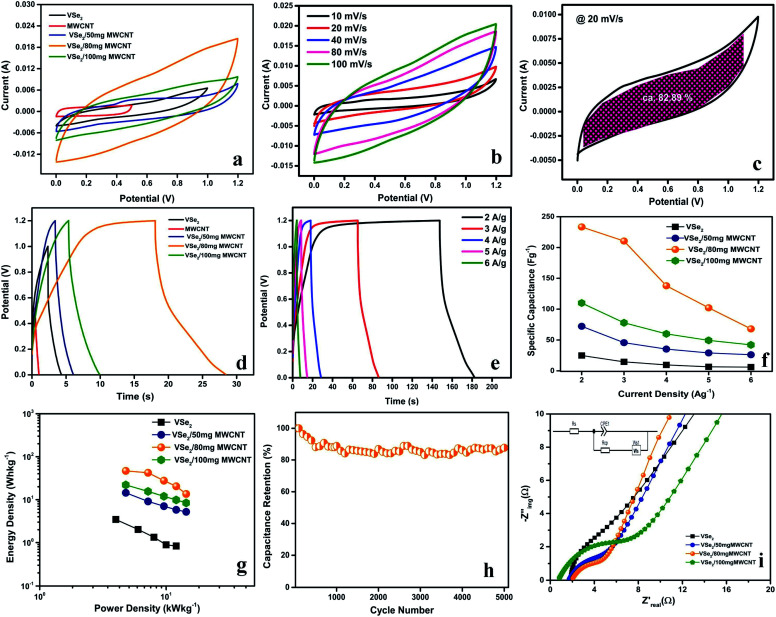
(a) Comparative CVs of VSe_2_, MWCNT and VSe_2_/MWCNT hybrid electrodes at a scan rate of 100 mV s^−1^, (b) CVs of VSe_2_/80 mg MWCNT at varying scan rates, (c) capacitive and diffusion controlled charge storage for CV at a scan rate of 20 mV s^−1^ CVs of VSe_2_/80 mg MWCNT, (d) GCD of VSe_2_, MWCNT and VSe_2_/MWCNT at a current density of 4 A g^−1^, (e) GCD of VSe_2_/80 mg MWCNT at different current densities for, (f) specific capacitance *vs.* current density plot, (g) Ragone plot of VSe_2_, and VSe_2_/MWCNT hybrid electrodes (h) cyclic stability of VSe_2_/80 mg MWCNT during 5000 GCD cycle and (i) Nyquist plot of VSe_2_, and VSe_2_/MWCNT hybrid electrodes (inset) equivalent circuit.

Fig. 2c shows the voltage–current profile of VSe_2_/80 MWCNT composite at a scan rate of 20 mV s^−1^ and the shaded region accounts for the capacitive controlled region. The dominance of capacitive contribution for the charge storage in VSe_2_/80 mg MWCNT electrodes is evident from Fig. 2c. In VSe_2_/80 mg MWCNT, the capacitive contribution is found to be around 82.89%. A gradual increase in capacitive contribution for VSe_2_/80 mg MWCNT is found with increasing scan rate, at a higher scan rate of 100 mV s^−1^, segregation of capacitive contribution over diffusion is around 92.75% (Fig. S8a of ESI†). We can take an assumption that the majority of the charge storage phenomena in VSe_2_/80 mg MWCNT electrode is due to the capacitive contribution, whereas fast intercalation of K^+^ ions contributes towards the diffusion process.

With the help of Trasatti method, the charge storage mechanism inside and outside of the VSe_2_/80 mg MWCNT electrode can be further explained (Fig. S8b and c of ESI†). From the *y*-intercept of 1/*q vs. υ*^1/2^ (where *υ* = 0), the total charge stored (*Q*_total_) can be calculated and charge stored on the outer surface of the electrode (*Q*_outer_) can be obtained from the *y*-intercept of *q vs. υ*^−1/2^ (where *υ* = ∞).^41^ The total charge stored in the electrode and on the surface of the electrode is calculated to be 217.86 C g^−1^ and 54.23 C g^−1^ respectively and the charge stored inside the electrode is 163.63 C g^−1^. This upholds the faradaic component in the charge storage operation along with the dominance of capacitive contribution. The sharp increase in the intensity of (002) peak and diminution of highly intense (011) peak observed in the XRD spectrum of VSe_2_/80 mg MWCNT after 5000 cycles, explain a possible intercalation effect of the cation in the gap between the VSe_2_ layers. This observation further provides the insights to the observed enhanced pseudocapacitive energy storage performance arising from the intercalation of cations (Fig. S8d ESI†).^25^

Fig. 2b, S6b and d† and f illustrate the GCD curves of VSe_2_ and VSe_2_/MWCNT hybrids at varying current densities. The energy storage performance of VSe_2_/MWCNT is found to be very much superior compared to the pristine VSe_2_ sheets. VSe_2_/80 mg MWCNT shows superior supercapacitor performance than that of all other electrodes with a specific capacitance of 233.33 F g^−1^ at a current density of 2 A g^−1^Fig. 2f. The dominance of MWCNT content and its aggregation in the VSe_2_/100 mg MWCNT composite can be accounted for the truncated electrochemical performance compared to VSe_2_/80 mg MWCNT. The coulombic efficiency of VSe_2_/80 mg MWCNT increases with increase in current density (Fig. S9 of ESI†). At lower current density the electrode might undergo some parasitic reactions, which may be originated from the diffusion and responsible for the low coulombic efficiency of VSe_2_/80 mg MWCNT at lower current density.^42–44^ The VSe_2_/80 mg MWCNT possesses high cyclic stability with 87% of capacitive retention after 5000 GCD cycles and a 95% of coulombic efficiency which provides high reversibility and higher power operation (Fig. 2f).^45^ The interconnection of 2D VSe_2_ sheets and 1D MWCNT not only enhances the aspect ratio of the stored ions but gives a shorter ion transport pathways to the inner side of the material. Besides, VSe_2_/80 mg MWCNT exhibits a much small *iR*_drop_ compared to the pristine VSe_2_ and this leads to the reduction of equivalent series resistance (ESR) from 20.96 Ω cm^2^ to 12.67 Ω cm^2^. The reduction observed in ESR is probably due to the enhancement in ionic conductivity of VSe_2_/80 mg MWCNT.^46^

A comparative Ragone plot of VSe_2_ and VSe_2_/MWCNT hybrids is shown in Fig. 2g. VSe_2_/80 mg MWCNT exhibits an energy density of 46.66 W h kg^−1^ at a high power density of 4.8 kW kg^−1^ and retains 13.6 W h kg^−1^ of its energy density at a power density of 14.4 kW kg^−1^ which further underlines the high power operation of VSe_2_/80 mg MWCNT. All the VSe_2_ based hybrid materials we discussed above possess an excellent power capability. From the Ragone plot, it is evident that the high power density in the composites came from the metallic nature of VSe_2_ and the addition of nanotubes enhances the energy density of the composites. The VSe_2_/80 mg MWCNT hybrid possesses high cyclic stability with 87% of capacitive retention after 5000 GCD cycles (Fig. 2h).^45^ A comparison of delivering a high energy density at a higher power density in TMD based devices along with the present work is given in Table 1; which further underlines the superior performance of VSe_2_/80 mg MWCNT.

**Table 1:** Comparative table on the energy storage performance of existing literature on TMDs based supercapacitor devices and the present work

Active material	Type of supercapacitor	Energy density	Cyclic stability%/cycles	Ref.
Flower-like MoS_2_/GNS	Asymmetric	78.9 W h kg^−1^ at 284.1 W kg^−1^	90/5000	49
MoS_2_	Symmetric	34.0 W h kg^−1^ at 333.3 W kg^−1^	81.6/3000	50
NiSe@MoSe_2_	Asymmetric	32.6 W h kg^−1^ at 415 W kg^−1^	91.4/5000	51
MoSe_2_/graphene	Asymmetric	26.6 W h kg^−1^ at 0.8 kW kg^−1^	88/3000	52
WS_2_	Symmetric	31.9 W h kg^−1^ at 333.3 W kg^−1^	77.4/3000	50
WS_2_/rGO	Symmetric	49 W h kg^−1^		53
WSe_2_/rGO	Symmetric	34.5 W h kg^−1^ with 400 W kg^−1^	98.7/3000	54
SnS_2_/GCA	Asymmetric (sodium hybrid capacitor)	108.3 W h kg^−1^ at 130 W kg^−1^	68.4% after 1500 cycles at 1 A g^−1^	55
NiSe_2_ spheres	Asymmetric	35.2 W h kg^−1^ at 749.3 W kg^−1^	97.3/10 000	56
CoSe_2_	Asymmetric	32.2 W h kg^−1^ at 1914.7 W kg^−1^	94.5/5000	57
CoS_2_	Symmetric	11.8 W h kg^−1^ at 0.3 kW kg^−1^		58
1T′-MoTe_2_	Asymmetric	56.4 W h kg^−1^ at 800 W kg^−1^		59
TiS_2_/VACNT	Symmetric	60.9 W h kg^−1^	>95/10 000	60
VS_2_	Symmetric	25.9 W h kg^−1^ at 1.5 kW kg^−1^	89/6000	61
VS_2_/MWCNTs	Symmetric	42 W h kg^−1^ at 2.8 kW kg^−1^	93.2/5000	37
VSe_2_/rGO	Symmetric	212 W h kg^−1^ at 0.9 kW kg^−1^	81/10 000	25
**VSe**_**2**_ **/MWCNTs**	**Symmetric**	**46.66 W h kg** ^**−1**^ **at 4.8 kW kg** ^**−1**^	**87/5000**	**This work**

With the help of electrochemical impedance spectroscopy (EIS) measurement, the charge transfer property and resistivity of these materials have been explored. The Nyquist plots for the pristine VSe_2_ and VSe_2_/MWCNT composites with their Randles equivalent circuit (inset) are shown in Fig. 2i and S7c† EIS spectrum of MWCNT has also been shown. All the composites show far better charge transfer as compared to the pristine VSe_2_. However, VSe_2_/80 mg MWCNT exhibits higher charge transfer than any other composites which further proves its excellent capacitance.^45^ The synergistic mechanism between VSe_2_ sheets and multi-walled carbon nanotube in VSe_2_/80 mg MWCNT improves the electronic conductivity and diminishes resistivity which further sheds light on the enhanced power operation of this electrode material.^34,47,48^ The charge storage mechanism and reduced redox kinetics of VSe_2_/80 mg MWCNT can be further understood by its fast charge transfer kinetics.

### 3.2 Theoretical study of VSe_2_/MWCNT hybrids using Density Functional Theory (DFT)

We have provided theoretical support using Density Functional Theory (DFT) simulations. Computational details are given in Section 2.4.

**Fig. 3:**
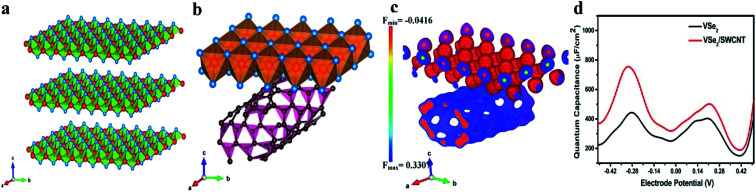
(a) DFT optimized structure of VSe_2_ layers; red and blue spheres represent vanadium and selenium atoms respectively, (b) optimized structure for VSe_2_/SWCNT hybrid; red, blue and purple spheres represent vanadium, selenium and carbon atoms respectively. (c) Charge density distribution plot for charge density difference between VSe_2_/SWCNT and VSe_2_ for isovalue of 0.05*e*; charge gain region is shown by blue color and charge loss region by red color. (d) Variation of quantum capacitance with electrode potential for VSe_2_ and VSe_2_/SWCNT hybrid structure.

**Fig. 4:**
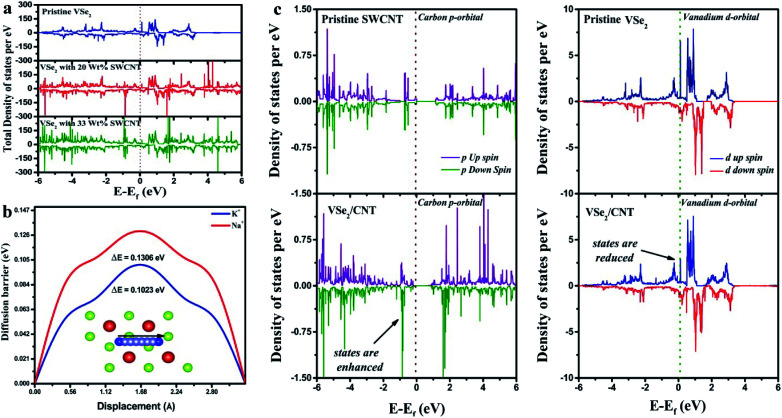
(a) Total density of states for VSe_2_ surface (upper plane) and VSe_2_/SWCNT hybrid surface with 20 wt% and 33 wt% SWCNT (lower panel); Fermi level is shown by dotted line. (b) Barrier energy comparison of K^+^ and Na^+^ ions on the surface of VSe_2_ (c) partial density of states for C 2p orbital for pristine SWCNT and VSe_2_/SWCNT (left); V 3d orbital for pristine VSe_2_ and VSe_2_/SWCNT (right).

First, we have generated (001) surface of VSe_2_ and performed geometry optimization. The relaxed structure of the (001) plane of VSe_2_ is displayed in Fig. 3a. Then we have put single walled carbon nanotube (SWCNT) with chirality of (8,0) and allow the hybrid structure to relax. The hybrid VSe_2_–SWCNT is depicted in Fig. 3b. The separation between VSe_2_ and SWCNT is around 3 Å. Here we mention that for theoretical simulations we have considered only single walled carbon nanotube (SWCNT) as taking more layers connected by weak van der Waal's interactions is computationally expensive and does not provide much additional information. To get the electronic properties of the hybrid structure, we have computed the density of states (DOS). The DOS for pristine VSe_2_, VSe_2_/20 wt% of SWCNT (∼VSe_2_/50 mg MWCNT) and VSe_2_/33 wt% SWCNT (∼VSe_2_/80 mg MWCNT) are shown in Fig. 4a. We can notice the enhancement of states near Fermi level in the hybrid structure compared to pristine VSe_2_. The enhancement is stronger with the increase in SWCNT content. Electronic structures with more states near Fermi level may point towards an increase in conductivity of VSe_2_ when it is hybridized with SWCNT which supports our experimental observations. Fig. 4c depicts the partial density of states of C 2p orbital and V 3d orbital for pristine SWCNT, pristine VSe_2_ and for the hybrid structure. We can observe that the states of V 3d near Fermi level gets reduced in the hybrid structure compared to pristine VSe_2_. Also, the states of C 2p orbital near Fermi level gets enhanced for the hybrid structure compared to pristine SWCNT. The reduction of states of V 3d orbitals and increase in states for C 2p orbitals near Fermi level for the hybrid structure indicates a possible charge transfer between V 3d orbital and C 2p orbitals. To visualize the charge transfer qualitatively, in Fig. 3c, we have plotted the charge density distribution for charge density difference between VSe_2_/SWCNT and VSe_2_ for isovalue of 0.05*e*. Charge gain region is shown by blue color and charge loss region by red color. We can see more blue color iso-surface in SWCNT indicating the charge gain by C 2p orbital from V 3d orbital. This is consistent with the charge transfer as seen from the analysis of the partial density of states presented in Fig. 4c.

From the density of states, we have computed the quantum capacitance using the relation^62^5
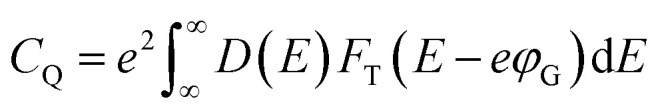
where the parameters are *D*(*E*) = density of states, *φ*_G_ = electrode potential, *F*_T_(*E*) = thermal broadening function.

The thermal broadening function is given by6
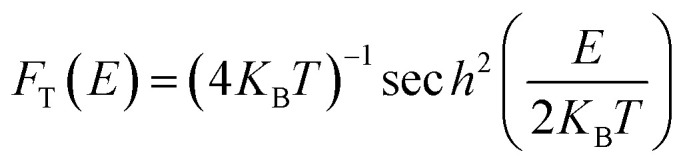


Fig. 4d shows the variation of quantum capacitance with the electrode potential for pristine VSe_2_ surface and the hybrid VSe_2_/SWCNT surface. We can notice that the quantum capacitance is higher for the hybrid VSe_2_/SWCNT compared to pristine VSe_2_ surface. Higher quantum capacitance for the hybrid structure qualitatively justifies the better supercapacitor performance of the hybrid structure. Here we clarify that quantum capacitance is significant for low dimensional system and in the experiment we measure the total capacitance which includes both quantum capacitance and electrical double layer capacitance.

K_2_SO_4_ aqueous electrolyte is used for the electrochemical energy storage of the present system. We have computed the average voltage of the system for different concentrations of K^+^ ions using the eqn (7) (ref. 63)7
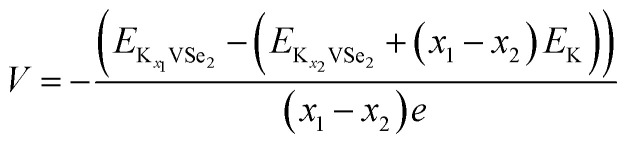
Here *E*_K_ and K_*x*_VSe_2_ represent the energy of K^+^ per ion and total energy of K_*x*_VSe_2_ per unit formula, respectively. We have chosen three different concentrations for K^+^ ions with *x*_1_, *x*_2_, *x*_3_ are 0.03333, 0.13333 and 0.26666 respectively. The computed average voltage for different concentration of K^+^ ions is listed in Table S1 of ESI.† The voltage range is consistent with the theoretical predictions for a similar system in the literature.^33^ In Fig. S10 of ESI,† we have plotted the energy of the system for different concentration of K^+^ ions.

As the mobility of the electrolyte ions increases, the system exhibits better super capacitance performance. The mobility of the ion is inversely proportional to the barrier potential offered by the system. We have computed the diffusion barrier of K^+^ and Na^+^ ions for the diffusion across the monolayer of VSe_2_. The computed diffusion barrier of K^+^ and Na^+^ ions are 0.1023 eV and 0.1306 eV respectively. We can see that the diffusion barrier for K^+^ ion is less compared to Na^+^ ions attributing higher mobility and higher charge transfer.

## 4 Conclusions

In summary, hybrid structures of metallic VSe_2_ and MWCNT were synthesised by a one-step hydrothermal method. Enhanced electrochemical energy storage performance was observed in all the composites compared to pristine VSe_2_. The VSe_2_/80 mg MWCNT hybrid electrode shows high cyclic stability, good energy density and an attractive power density in comparison with other hybrids. A predominant capacitive contribution over diffusion-controlled contribution in the energy storage mechanism was observed in all the hybrid electrodes of VSe_2_ and MWCNT. Synergistic effect of VSe_2_ and MWCNT elucidates the enhancement of electrochemical properties of VSe_2_/80 mg MWCNT and reduces the resistivity of the material. Besides the increase in interplanar spacing in VSe_2_ with the incorporation of MWCNT provides a favourable condition for the intercalation of K^+^ ions and improves the cyclic stability of the VSe_2_/80 mg MWCNT electrode in high power operation. We have presented the electronic properties and quantum capacitance of the hybrid structure VSe_2_/SWCNT to get theoretical insight for the enhanced charge storage performance of the hybrid structure. Enhanced states near Fermi level, charge transfer from V 3d orbital to C 2p orbitals and enhanced quantum capacitance of the hybrid structure provide theoretical justification for superior super capacitance performance of VSe_2_/SWCNT as observed in experiments. The low diffusion barrier of K^+^ ions (of K_2_SO_4_ electrolytes) compared to Na^+^ ions predicts higher mobility and better charge storage characteristics.

## Conflicts of interest

There are no conflicts to declare.

## Supplementary Material

RA-010-D0RA06773C-s001
