# Supplementary material for: The role of carbon nanotubes in enhanced charge storage performance of VSe2: experimental and theoretical insight from DFT simulations
Source: RSC Adv. 2020 Aug 27;10(53):31712–9. doi: 10.1039/d0ra06773c (PMC9056424; doi:10.1039/d0ra06773c)
Supplement: RA-010-D0RA06773C-s001 [file RA-010-D0RA06773C-s001.pdf]

Electronic Supplementary Material (ESI) for RSC Advances.
This journal is © The Royal Society of Chemistry 2020

Supplementary Material

The Role of Carbon Nanotubes for Enhanced Charge Storage Performance of VSe_2: Experimental and Theoretical Insight from DFT Simulations

Sree Raj K.A. [a], Afsal S. Shajahan [b] , Brahmananda Chakraborty [b], Chandra Sekhar Rout [a, *]*

[a] Centre for Nano and Material Sciences, Jain Global Campus, Jakkasandra, Ramanagaram, Bangalore-562112, India.

[b] High Pressure and Synchrotron Radiation Physics Division, Bhabha Atomic Research Centre, Trombay, Mumbai 400085, India.

Email: Corresponding author: *r.chandrasekhar@jainuniversity.ac.in; csrout@gmail.com, (CSR); brahma@barc.gov.in (BC)*

Electrochemical measurement details:

The specific capacitance (C_{sp}) was calculated from cyclic voltammograms by using the given equation:

$$C_{sp} = \frac{\int I(v)dv}{m * s * 2[V_f - V_i]} \quad (Fg^{-1}) \tag{1}$$

Where the integral part in the numerator gives the area under the CV curve, "m" is the mass of the active material, "s" is the scan rate, and $[V_f - V_i]$ is the potential window (V_f, V_i are the final and initial potential values respectively). From the charge/discharge curves, the specific capacitance of the material was calculated using the following equation:[1]

$$C_{sp} = 4 * \frac{I\Delta t}{m\Delta V} \text{ (Fg}^{-1}\text{)} \tag{2}$$

Where I is the discharge current, m is the mass of the sample deposited on the Ni foam surface and Δt is the discharge time.

The equivalent series resistance (ESR), Specific energy density(E_d), and Specific Power density (P_d) can be calculated from the following equations

$$\text{ESR}= \frac{iR_{drop}}{2 * I} * A \quad (\Omega\text{cm}^2) \tag{3}$$

$$E_d = \left(\frac{1}{2} C_{sp} * (V_f - V_i)^2\right)/3.6 \text{ (Wh kg}^{-1}\text{)} \tag{4}$$

$$P_d = \frac{Ed}{t_d} * 3600 \text{ (W kg}^{-1}\text{)} \tag{5}$$

Where I(A) is the discharge current, A is the area of the electrode material and t_d (s) is the discharge time.

Results and Discussions

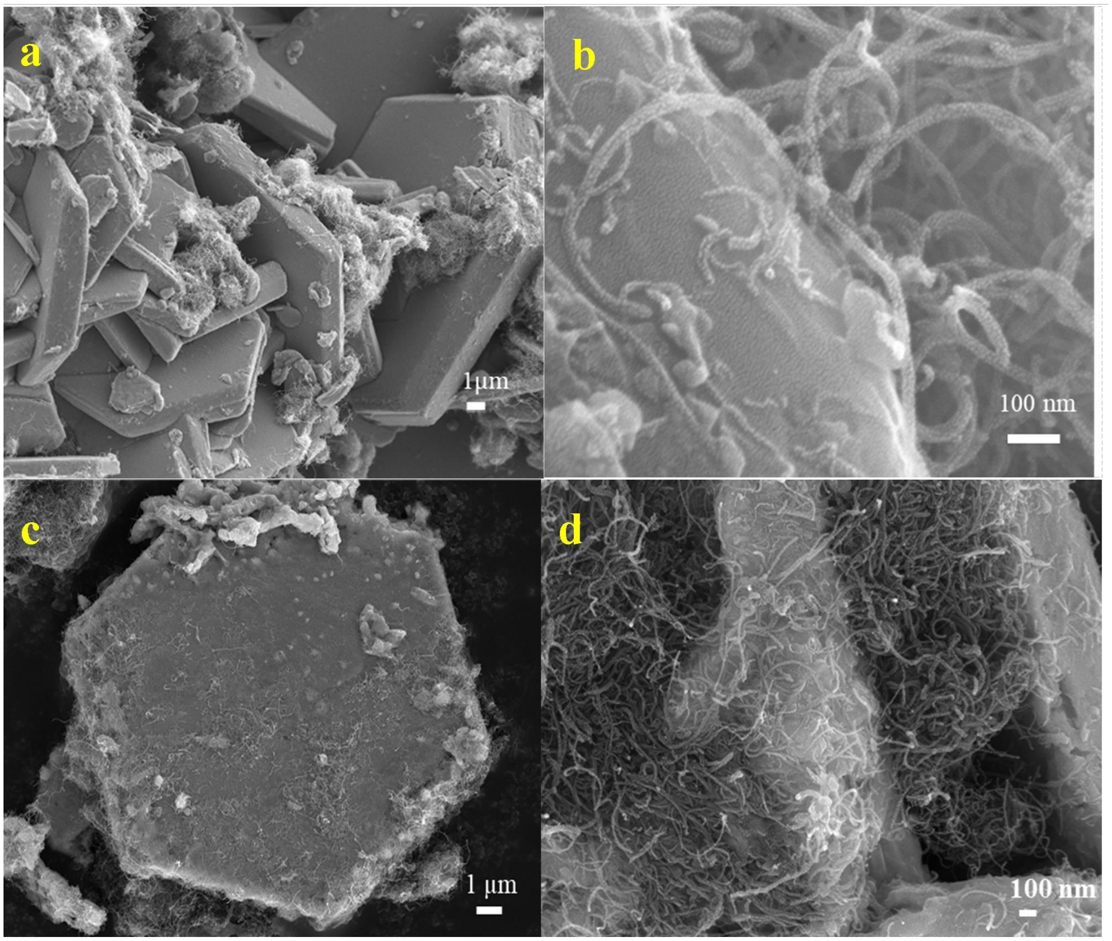

Figure S1. FESEM images for (a) low magnification and (b) high magnification of VSe_2/50mg MWCNT, (c) low magnification and (d) high magnification VSe_2/100mg MWCNT composite

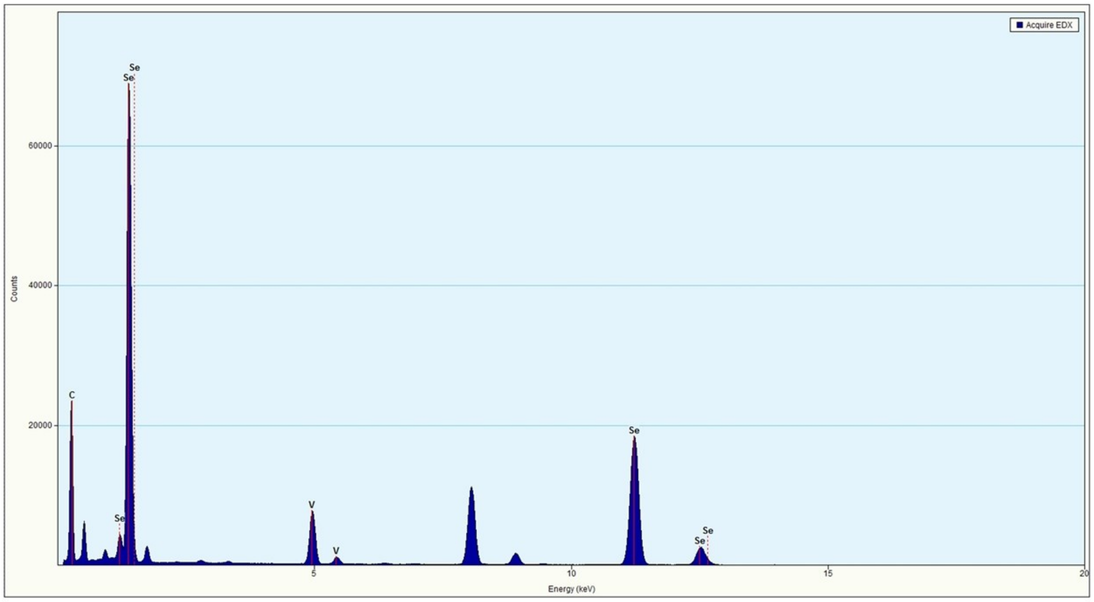

Figure S2. EDS spectrum of VSe_2/80mg MWCNT

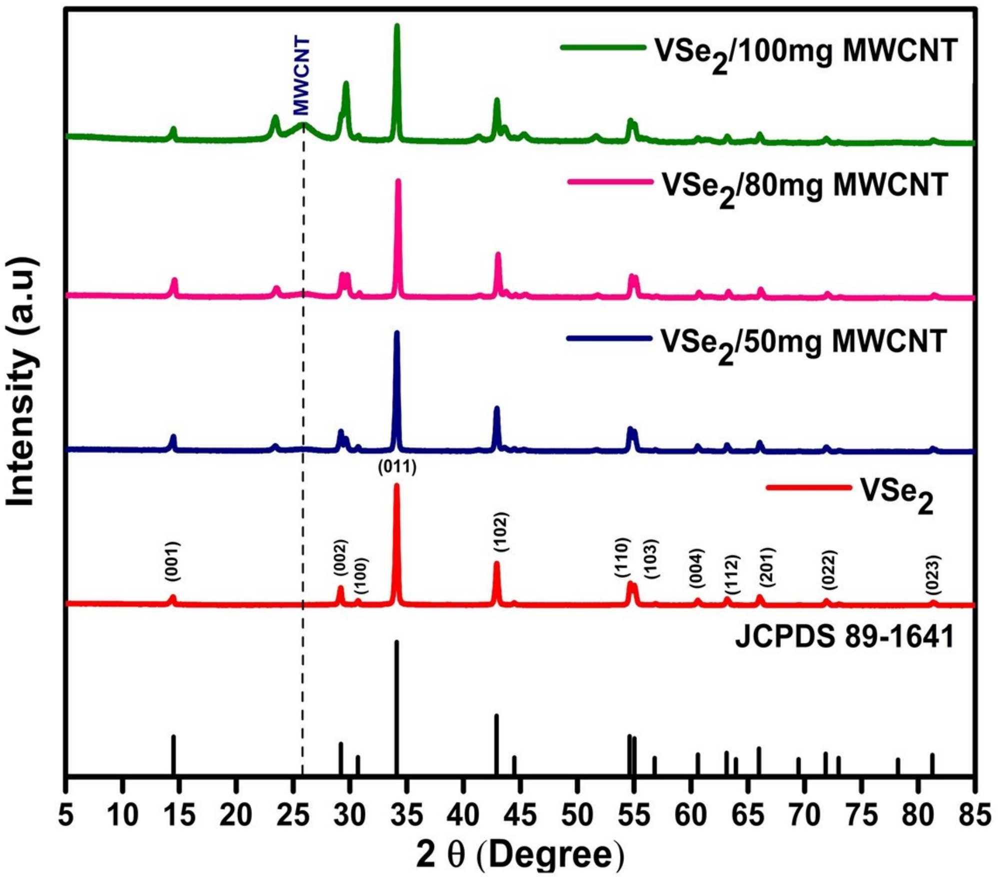

Figure S3. XRD spectrum of pristine metallic VSe_2 and all VSe_2/MWCNT composites

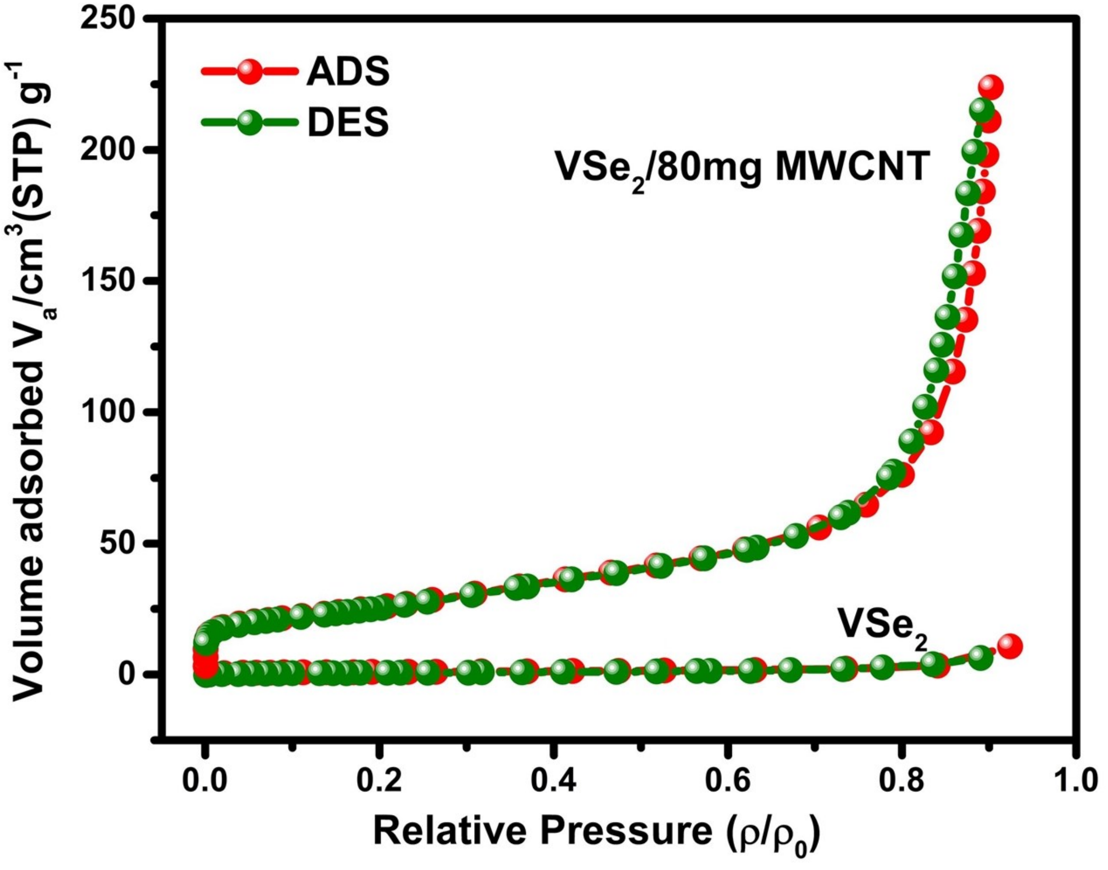

Figure S4. BET surface area analysis by nitrogen adsorption/desorption isotherms of VSe_2 and VSe_2/80mg MWCNT composite.

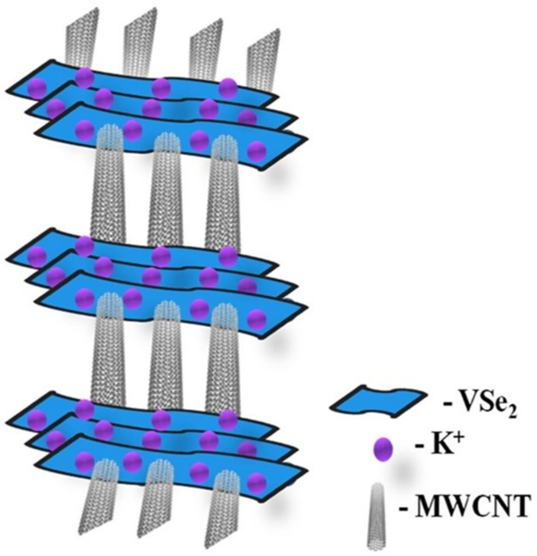

Figure S5. Schematic illustration of concatenated structure metallic VSe_2 layers and MWCNT with K^+ intercalation.

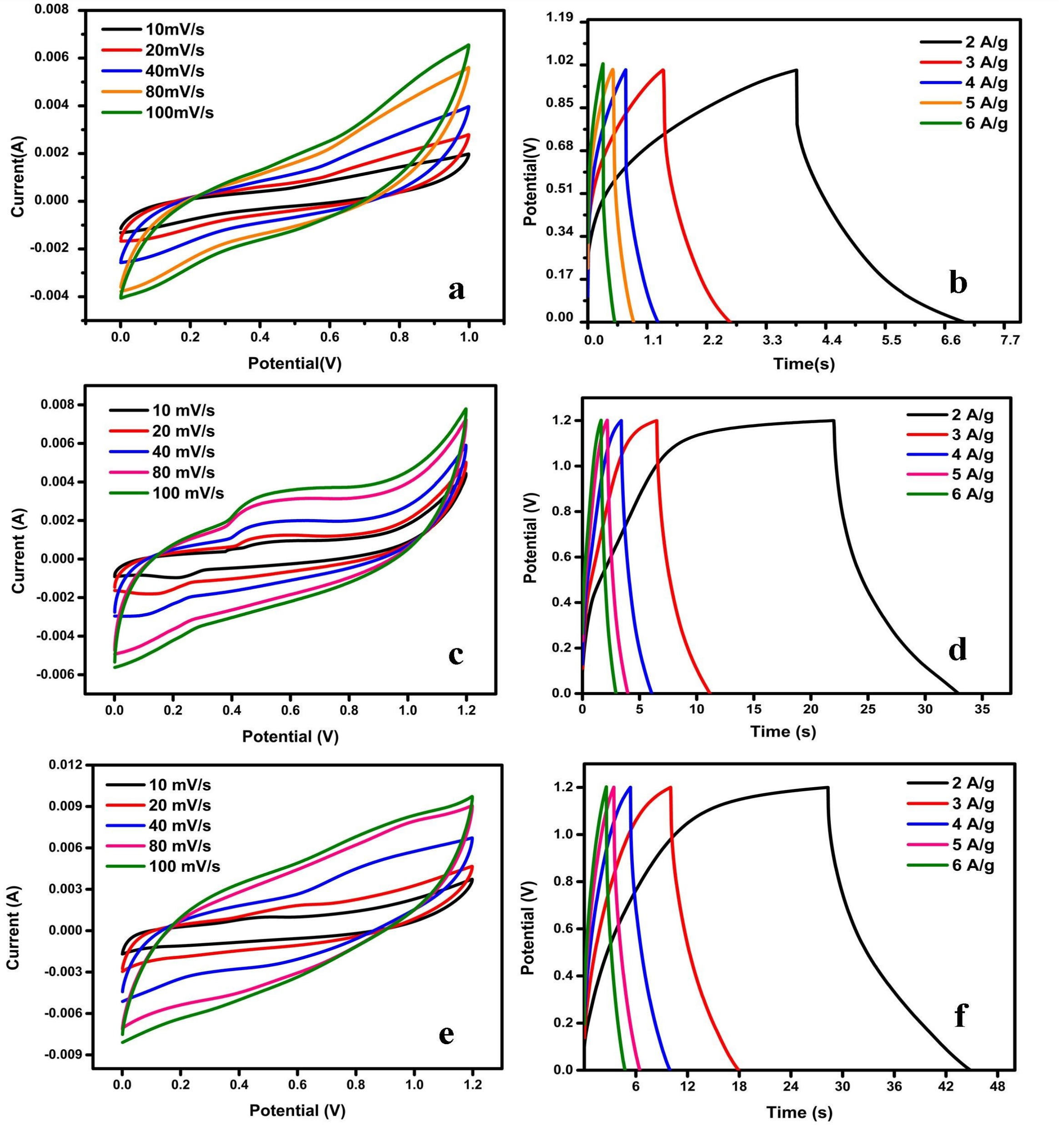

Figure S6. CVs of (a) VSe_2 (c) VSe_2/50mg MWCNT and (e) VSe_2/100mg MWCNT and at varying scan rates and GCD profiles of (b) VSe_2 (d) VSe_2/50mg MWCNT and (f) VSe_2/100mg MWCNT hybrid electrodes.

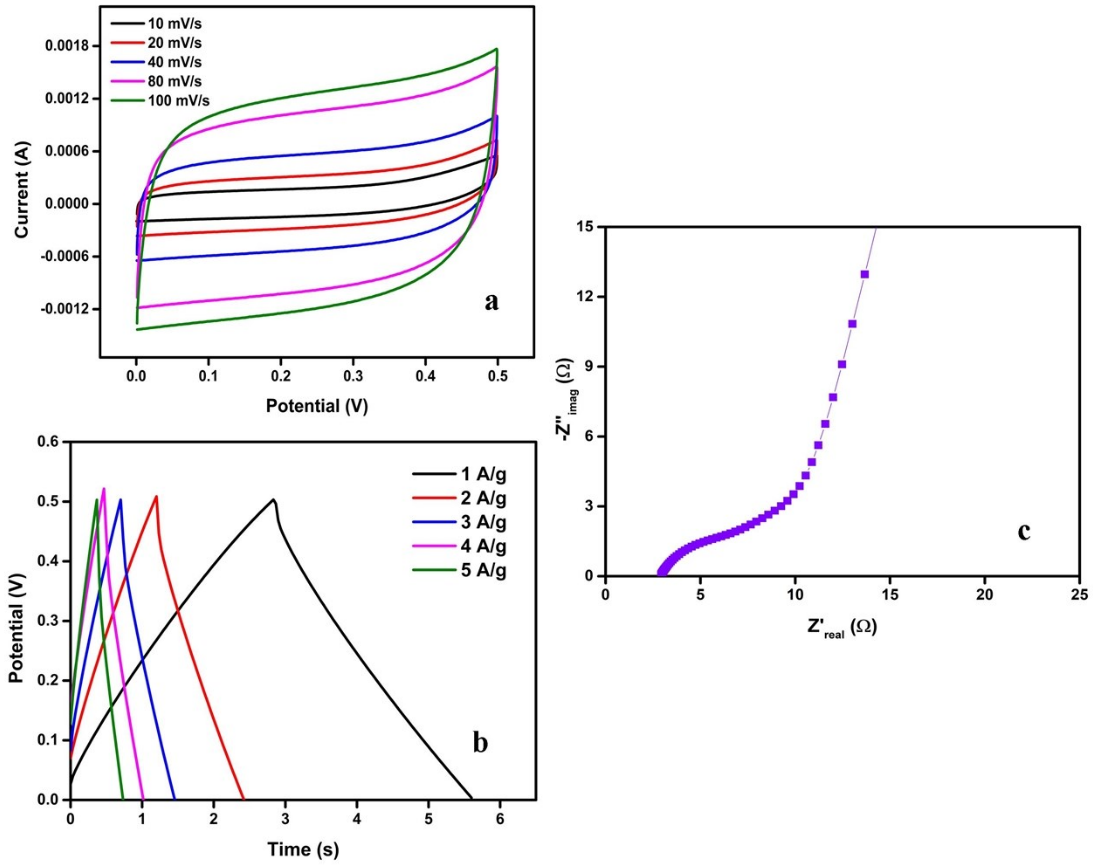

Figure S7. (a) CV of MWCNT at varying scan rates, (b) GCD of MWCNT at varying current densities and (c) Nyquist plot of MWCNT.

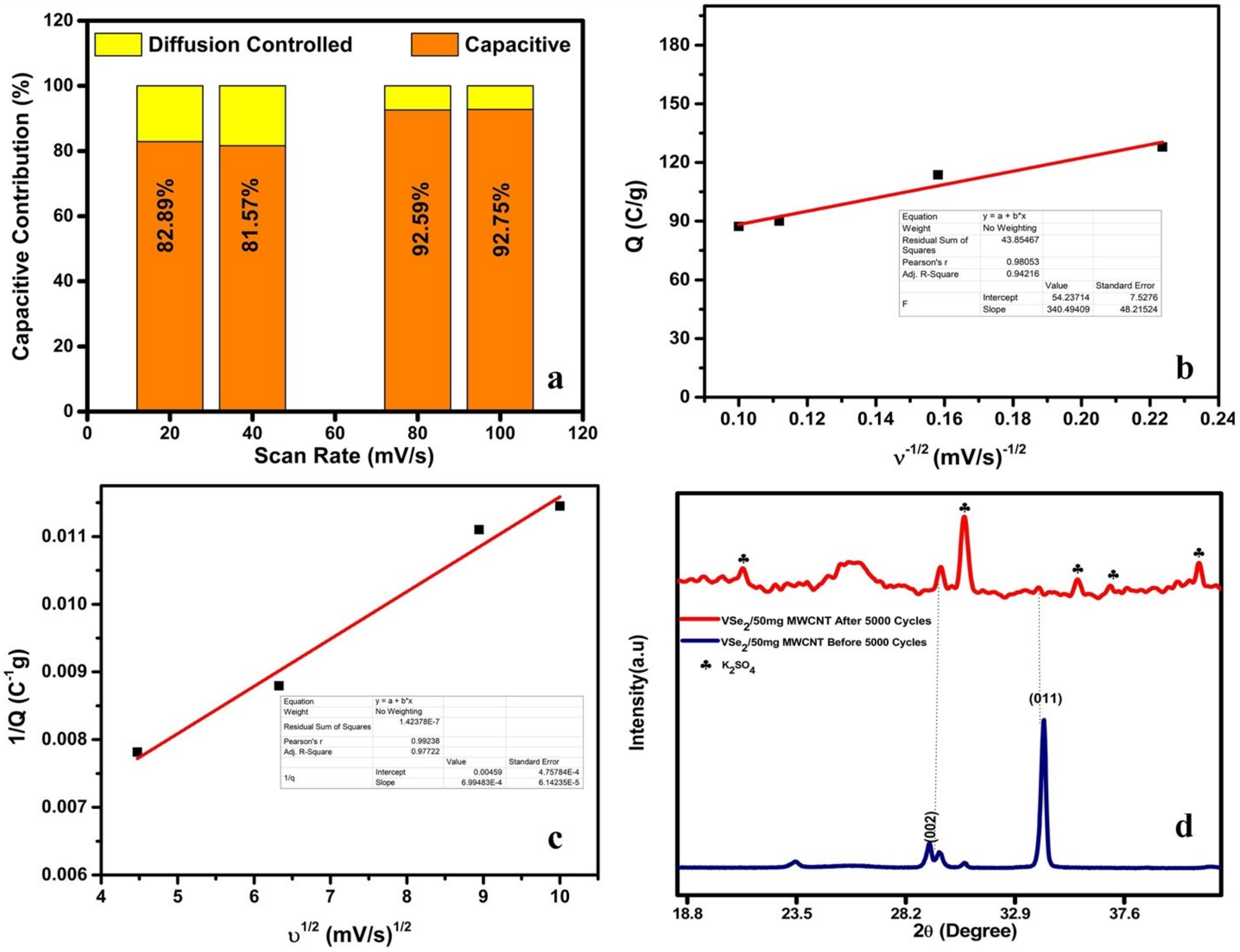

Figure S8. (a) segregation of capacitive and diffusion contributions at different scan rates, (b and c) Trasatti plots and (c) Comparison of XRD spectra for VSe_2/80mg MWCNT hybrid before (blue) and after (red) 5000 GCD cycles, extra peaks observed in the electrode after the measurement is due to K_2SO_4.

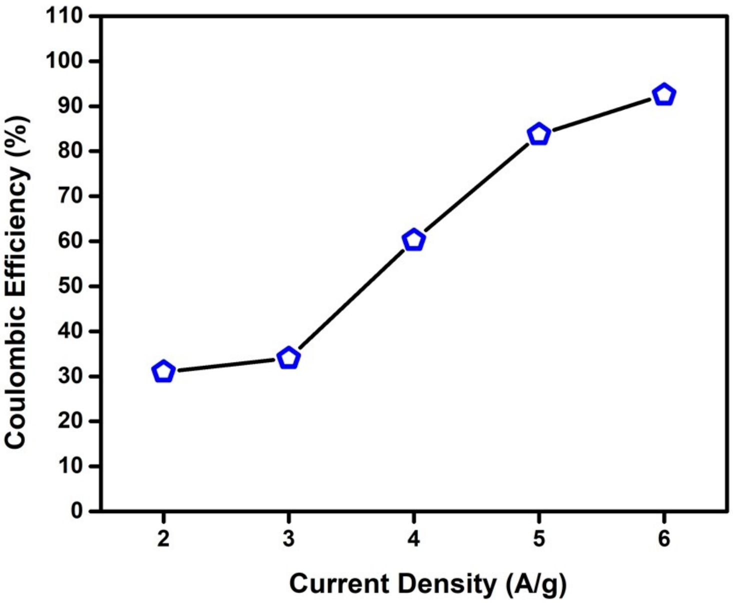

Figure S9. Coulombic efficiency Vs Current density plot of VSe_2/80mg MWCNT composite

$E_{K_{x_1}VSe_2}$ (eV)	$E_{K_{x_2}VSe_2}$ (eV)	V_{12} (V)
-18.1333333	-18.4200000	1.25
-18.0733333	-18.4200000	1.91
-18.0733333	-18.1333333	2.80

Table S1. Calculated average voltages as a function of K^+ ion concentration in VSe_2. $E_{K_xVSe_2}$ represent and total energy of K_xVSe_2 per unit formula , where x are 0.03333,0.13333,0.26666 respectively.

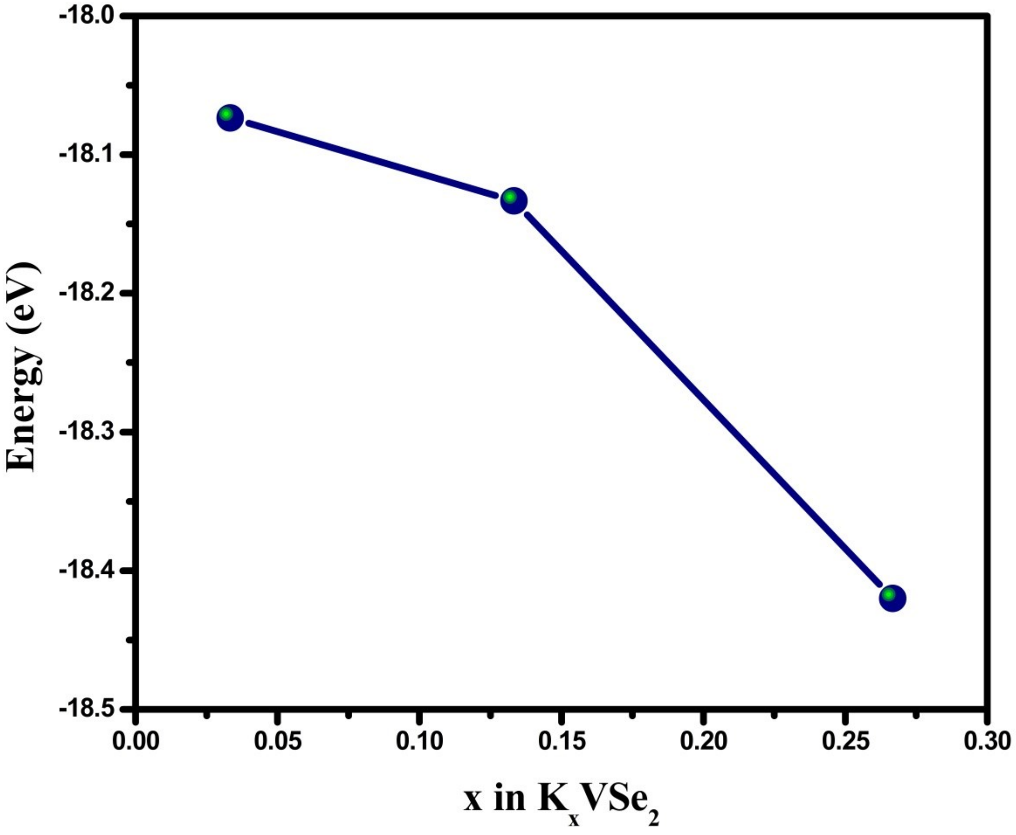

Figure S10. Total energy of K_xVSe_2 per unit formula with respect to the variation of K^+ concentration.

References

1 W. Zhang, B. Zhao, Y. Yin, T. Yin, J. Cheng, K. Zhan, Y. Yan, J. Yang and J. Li, *J. Mater. Chem. A*, 2016, **4**, 19026–19036.
